# Utilizing harmonization and common surveillance methods to consolidate 4 cohorts: the Western Alaska Tribal Collaborative for Health (WATCH) study

**DOI:** 10.3402/ijch.v72i0.20572

**Published:** 2013-05-02

**Authors:** Kathryn R. Koller, Abbie W. Wolfe, Jesse S. Metzger, Melissa A. Austin, Scarlett E. Hopkins, Cristiane Kaufmann, Stacey E. Jolly, Sven O.E. Ebbesson, Jason G. Umans, Barbara V. Howard, Bert B. Boyer

**Affiliations:** 1 Division of Community Health Services, Alaska Native Tribal Health Consortium, Anchorage, AK, USA; 2 Center for Behavioral Health, University of Alaska Anchorage, Anchorage, AK, USA; 3 Department of Epidemiology, University of Washington, Seattle, WA, USA; 4 Center for Alaska Native Health Research, University of Alaska Fairbanks, Fairbanks, AK, USA; 5 General Internal Medicine, Cleveland Clinic Medicine Institute, Cleveland, OH, USA; 6 Norton Sound Health Corporation, Nome, AK, USA; 7 MedStar Health Research Institute, Hyattsville, MD, USA; 8 Georgetown-Howard Universities Center for Clinical and Translational Science, Washington, DC, USA

**Keywords:** Alaska Native, cardiovascular disease, type 2 diabetes, cohort study, statistical power, risk factors

## Abstract

**Background:**

According to health status reports, chronic disease prevalence appears to be rising in western Alaska Native (AN) people, and accurate population-based data are needed. Four cohort studies of western AN people were conducted in the Norton Sound and Yukon-Kuskokwim regions, but none have been large enough to allow reliable estimates of rates of chronic diseases and evaluate their risk factors.

**Objective:**

In this article, the methods used to combine 4 major cohort studies of rural western AN people are described and the benefits and challenges encountered in combining data and standardizing surveillance methods for these studies are discussed.

**Design:**

Tribal permission was obtained for each cohort study and the consolidated study. Data from baseline exams were directly combined or harmonized into new variables. Common surveillance methods were developed and implemented to identify incidence and risk factors for cardiovascular disease (CVD) events and type 2 diabetes.

**Results:**

A cohort of 4,569 western AN participants (2,116 men and 2,453 women), aged 18–95 years, was established to study CVD and diabetes prevalence. Prospective surveillance data over an average 6.7-year follow-up can now be used to study CVD and diabetes incidence and associated risk factors in a subset of 2,754 western AN participants (1,218 men and 1,536 women) who consented to initial surveillance.

**Conclusions:**

The combined cohort provides statistical power to examine incidence rates and risk factors for CVD and diabetes and allows for analyses by geographic region. The data can be used to develop intervention programmes in these populations and others.

Reports in the mid-1950s suggested a low prevalence of cardiovascular disease (CVD) and type 2 diabetes mellitus (DM2) in western Alaska Native (AN) people living in remote, rural communities of the Norton Sound and Yukon-Kuskokwim regions of Alaska (1). Recent data suggest that chronic disease prevalence is rising (2–5).

Although western AN people are culturally, linguistically, and geographically diverse, the Norton Sound communities are primarily Inupiat and the Yukon-Kuskokwim communities are primarily Yup'ik ethnicity (6). Further, these regions include the Central Yup'ik, Cup'ik, Inupiat and Siberian Yup'ik ethnic subgroups (7).

Four cohort studies were conducted in these regions; however, none was large enough to provide reliable data on incidence of chronic disease or to examine associations between risk factors and chronic diseases. The Western Alaska Tribal Collaborative for Health (WATCH) study combined the 4 major cohort studies: the Alaska–Siberia Project (ASP), the Center for Alaska Native Health Research (CANHR), the Alaska Education and Research Toward Health (EARTH) and the Genetics of Coronary Artery Disease in Alaska Natives (GOCADAN).

Overall goals were to create a large representative cohort to describe the prevalence and incidence of CVD and DM2 and examine risk factors influencing these diseases in western AN people. This article describes the original study samples, methods used to consolidate baseline data, and common methods used to identify incident CVD events and DM2 in a representative subset.

## Methods

The WATCH protocol was approved by the University of Alaska Fairbanks, the MedStar Health Research Institute, and the Alaska Area institutional review boards (IRBs). Tribal approval was granted by the Alaska Native Tribal Health Consortium, the Norton Sound Health Corporation, and the Yukon-Kuskokwim Health Corporation.

### Original studies

Modelled after the design and methods of the Strong Heart Study (8), the ASP was initiated in 4 communities in Norton Sound (9; Sven O.E. Ebbesson, primary investigator) in 1994 to characterize CVD and DM2 prevalence and risk factors in the region. A total of 449 Inupiat or Yup'ik participants were initially examined in 1994 (9). A second enrolment phase conducted in 1998 added baseline data for another 135 participants (10). Excluding non-western AN participants, the sample size for ASP was 584 participants (Table I).

**Table I:** Demographic characteristics of participants in original studies and the total WATCH cohort

Characteristic	ASP	CANHR	EARTH	GOCADAN	WATCH
Total *N*	584	1,157	1,493	1,335	4,569
Median age (years)	43	36	38	41	39
Age range (years)	25–91	18–94	18–86	18–95	18–95
Gender, *N* (%)					
Men	271 (46)	542 (47)	699 (47)	604 (45)	2,116 (46)
Women	313 (54)	615 (53)	794 (53)	731 (55)	2,453 (54)

ASP=Alaska–Siberia Project; CANHR=Center for Alaska Native Health Research; EARTH=Education and Research Toward Health; GOCADAN=Genetics of Coronary Artery Disease in Alaska Natives.

The CANHR studies are population-based observational studies to improve understanding of risk factors for obesity, DM2, and CVD (11); Gerald V. Mohatt and Bert B. Boyer, primary investigators). The cohort is composed predominantly of Yup'ik participants living in 11 communities in the Yukon-Kuskokwim region. Enrollees participated in at least 1 of 3 projects examining genetic, nutritional, and cultural–behavioural factors related to the development of chronic diseases. This cohort contributed 1,157 participants of Yup'ik/Cup'ik ancestry to the WATCH cohort (Table I).

The EARTH study is a community-based observational investigation of chronic disease risk factors (12); Anne P. Lanier, primary investigator) in a cohort of American Indian/AN people living in 3 U.S. regions: the southwest, the northern plains, and Alaska. The 3,828 AN participants were recruited during 2004–2006 from southeastern, south central, and southwestern Alaska. Of the Alaska EARTH study participants, 1,493 predominantly Yup'ik participants in 17 Yukon-Kuskokwim communities were included in WATCH (Table I).

GOCADAN is a population-based prospective cohort study with enrolment initiated in 2000–2004 (13; Barbara V. Howard, Jason G. Umans, Anthony Comuzzie, and Sven O.E. Ebbesson, primary investigators). A total of 1,214 participants were recruited in 9 predominantly Inupiat communities in the Norton Sound region (13). Follow-up examinations took place in 2006–2010 (80% of those examined at baseline) and added 160 newly recruited participants. Excluding non-Native participants, and including both enrolment phases, the GOCADAN study collected baseline data for 1,335 western AN participants (Table I).

### WATCH organization

Three committees were developed for WATCH.

#### WATCH Tribal Coordinating Committee

The Tribal Coordinating Committee was established to maintain the community-based participatory methods promoted in each original study and to provide community input and oversight. This committee is composed of tribal leaders from the Alaska Native Tribal Health Consortium, Norton Sound Health Corporation, and Yukon-Kuskokwim Health Corporation boards and the Alaska Native Health Board, which represents AN people statewide (14). It convened annually to receive updates, review findings, guide data dissemination, and provide research direction.

#### WATCH steering committee

The steering committee, comprised of the primary and co-investigators from the original 4 studies and the WATCH operations leader, was designed to oversee WATCH operations. This committee maintained continuous links to tribal communities through the Tribal Coordinating Committee. The steering committee continues to provide a forum in which to obtain information about the original study designs and methods, access external collaborators, assure adherence to the study protocol, and analyze data.

#### WATCH operations committee

The operations committee was responsible for database construction and the WATCH data centre. This committee identified all similar baseline data collected by the original studies and recommended consolidation methods to the steering committee. Operations committee activities are coordinated by the operations leader, who works closely with the WATCH study coordinator, WATCH database manager, and key personnel familiar with the details of data collection and management in the original studies. The steering and operations committees continue to meet monthly by teleconference to review recommended procedures for data analysis and dissemination.

### WATCH database construction

**Fig. 1:**
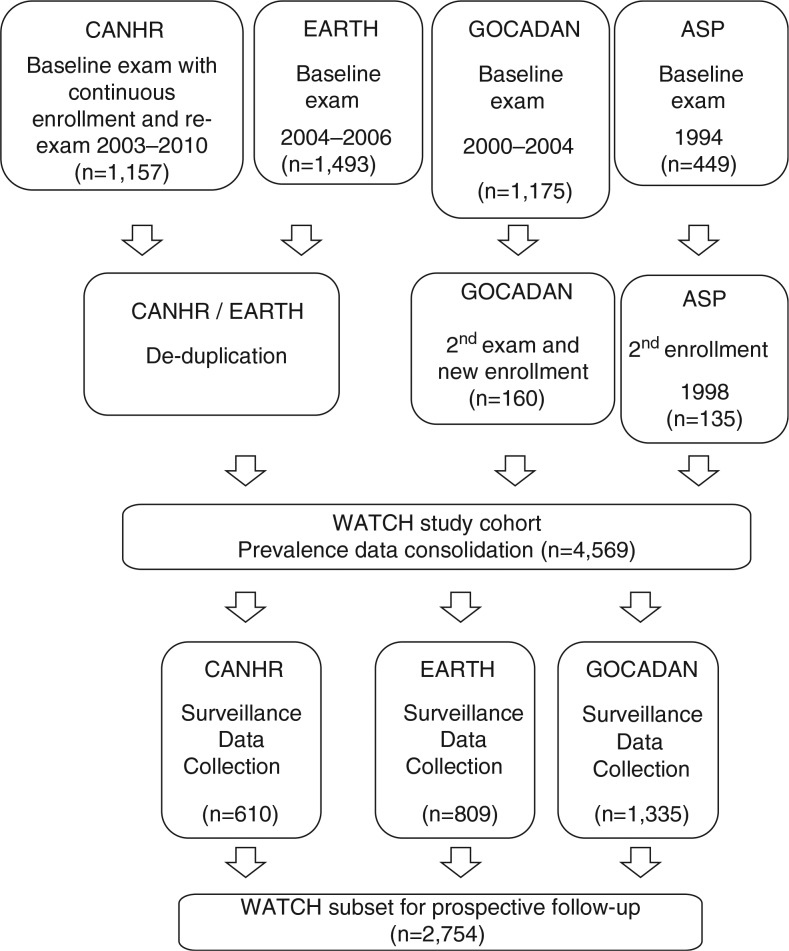
WATCH study data consolidation flow.

The WATCH data consolidation flow for both the prevalence and surveillance phases is shown in Fig. 1. For each original study, participants were assigned a study identifier (ID) and each study retained its own study ID key. Data managers ensured no cases were duplicated between studies (i.e. all cases were unique and were not included in more than 1 study). To maintain confidentiality, the operations committee replaced all originally assigned WATCH IDs with a second unique ID and retained the only key to the second set of WATCH IDs. For all medical record reviews, records were deidentified and a WATCH study ID was used.

The WATCH data set was created by: (a) identifying and consolidating baseline variables; (b) implementing consistent prospective surveillance methods to identify incident CVD events and DM2 cases; and (c) constructing a relational database to permit data export for statistical analyses.

### WATCH baseline data consolidation

The operations committee examined all data codebooks, operations manuals, and questionnaires from the 4 original studies, reviewed the literature comparing methods of data collection, and consulted with clinical and epidemiology experts. Data were first organized by similar topics, including clinical, anthropometric, and laboratory data; demographic and tobacco use information; self-reported medical and family history; self-perceived wellness; self-reported dietary intake; diet-derived nutrient intake; self-reported physical activity; and medical record-abstracted health history.

Variable keys were created for each topic area. Variables measuring the same property and having similar collection methods (e.g. height, weight, blood pressure, and waist and hip circumference) were combined directly. Variable keys were used to note variations in measurement techniques between studies. Variables measuring similar properties but obtained using dissimilar collection methods (e.g. data on tobacco use or physical activity) or requiring more than 1 data element (e.g. CVD events or prevalent diabetes defined by specific criteria), were harmonized into a newly created variable. Some variables (e.g. certain blood metabolites) collected by only 1 study and variables that were too dissimilar to be combined or harmonized were excluded.

#### Objective measurements

In general, clinical, anthropometric, and laboratory measurements were retained as continuous data. Minimal harmonization was necessary for these variables. The data were combined once all values were transformed to common measurement units.

#### Self-reported variables

Self-reported information on lifestyle and medical history was collected by all studies. Although questions and response choices varied, several demographic, tobacco use, personal health history, family history, self-perceived wellness, diet, and physical activity variables were combined or harmonized.

#### Medical history

A final set of WATCH disease prevalence variables was constructed. For prevalent CVD, the CANHR and EARTH studies reviewed all available medical records at baseline. The presence of diagnosis codes indicating CVD and cardiac procedures in these 2 studies was validated through supporting documents reviewed by trained research nurse abstractors. The ASP and GOCADAN studies limited baseline record review to participants who reported, or whose medical history suggested, a possible prior CVD event at baseline exam. GOCADAN gathered medical record information necessary for at least 2 physicians to adjudicate prior CVD events using standardized criteria. In the ASP cohort, a single physician reviewed medical records at baseline for prior events.

For the ASP, CANHR, and GOCADAN studies, reports of diagnosed diabetes, hypertension, and hypercholesterolaemia were confirmed by inspection of medications produced by participants at the baseline exam. For the EARTH and CANHR studies, these chronic conditions were *diagnosed* (present prior to baseline) if a diagnosis for the condition was noted in the participant's medical record. *Undiagnosed* diabetes, hypertension, and hypercholesterolaemia were defined in all 4 studies using baseline exam measurements in the absence of medication use or a pre-existing diagnosis in the medical record.

### WATCH incidence data and surveillance procedures

The aim of the WATCH surveillance was to use identical methods and to obtain complete ascertainment of all-cause mortality, CVD events, and incident DM2 occurring since baseline. Surveillance using standardized criteria included a review of all events that might be CVD- or DM2-related, followed by systematic adjudication of the participant medical records from the component studies. Follow-up ended in December 2010.

#### Mortality surveillance for total WATCH cohort

Deaths were identified using field contacts, newspaper obituary reviews, and vital statistics reports. Death certificates were requested and the causes of death were recorded. (Categories of fatal CVD events are available in Supplementary Table I.) In all cases of possible CVD deaths, records were reviewed for possible non-fatal CVD events during the year prior to the death, with separate adjudication packets created for each possible CVD event following the procedures for morbidity surveillance.

#### Morbidity surveillance in CANHR, EARTH and GOCADAN

Common procedures were adopted for the studies that obtained consent for prospective follow-up: CANHR, EARTH, and GOCADAN (Fig. 1).

Potential cases were identified by diagnoses or *International Classification of Diseases, Ninth Edition* (ICD-9) codes in the medical record indicating hospitalization for a wide range of possible CVD. Relevant information was deidentified, copied, and assembled into adjudication packets. All fatal and non-fatal events were adjudicated by 2 trained physicians, with a third physician consulted to resolve ambiguities. (Categories of non-fatal CVD events are available in Supplementary Table II.)

**Table II:** Demographic characteristics in the WATCH surveillance subset

Characteristic/risk factor	CANHR	EARTH	GOCADAN	WATCH
Total *N*	610	809	1,335	2,754
Median age (years)	40	41	41	41
Age range (years)	18–87	18–86	18–95	18–95
Gender, *N* (%)				
Men	248 (41)	366 (45)	604 (45)	1,218 (44)
Women	362 (59)	443 (55)	731 (55)	1,536 (56)

CANHR=Center for Alaska Native Health Research; EARTH=Education and Research Toward Health; GOCADAN=Genetics of Coronary Artery Disease in Alaska Natives.

Medical records from CANHR, EARTH, and GOCADAN were also reviewed for incident DM2, defined as: (a) having any laboratory results in follow-up exams (GOCADAN and CANHR) or the medical record that were within the diabetes range (fasting blood glucose ≥126 mg/dl, 2-hour oral glucose tolerance test blood glucose ≥200 mg/dl, or haemoglobin A1c≥6.5%) based on 2010 American Diabetes Association criteria (15); (b) having diabetes medications prescribed; or (c) having a diabetes diagnosis with supporting documentation. Each study collected data on the time and method of diagnosis, laboratory test results, and diabetes medications prescribed.

## Results

The combined WATCH study cohort with baseline data for prevalence consists of 4,569 study participants (Fig. 1). As shown in Table I, the median ages were 36–43 years, range 18–95 years. The proportion of women participating was similar across studies (53–55%).

Consent for prospective follow-up was not obtained by the ASP. In GOCADAN, all participants agreed to prospective follow-up. In CANHR and EARTH, signed consent to prospectively reviewed medical records was obtained from a subset of participants after initiation of the WATCH study (Table II).

**Fig. 2:**
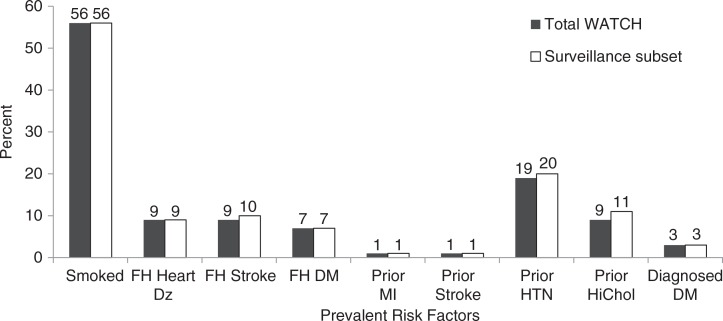
Comparison of select baseline characteristics in total WATCH cohort and follow-up subset. Note: Smoked=participants who currently or previously smoked cigarettes; FH=family history, DZ=disease, DM=type 2 diabetes, MI=myocardial infarction; HTN=hypertension; HiChol=high cholesterol.

**Fig. 3:**
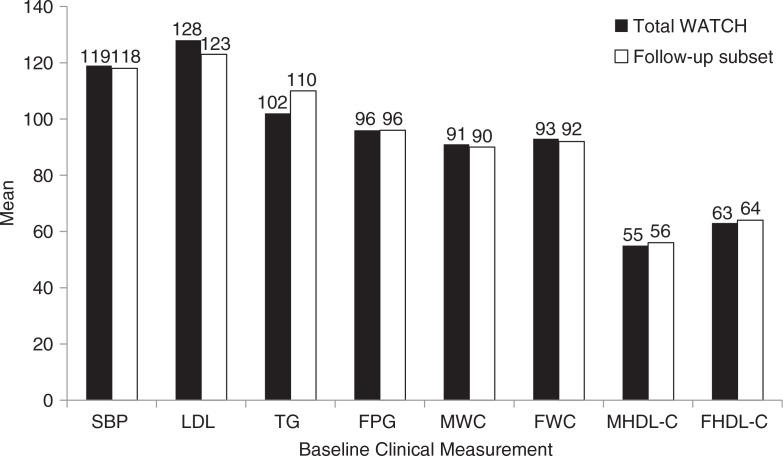
Comparison of means for baseline clinical measurements in total WATCH cohort and follow-up subset. Note: SBP=systolic blood pressure (in mmHg); LDL=low-density lipoprotein cholesterol, TG=triglycerides, FPG=fasting plasma glucose, M HDL-C=male high-density lipoprotein cholesterol, and F HDL-C=female high-density lipoprotein cholesterol (all in mg/dL); MWC=male waist circumference; and FWC=female waist circumference (both in centimetres).

The WATCH subset consenting to prospective surveillance (average 6.7 years of follow-up, range 4.0–14.4 years) consisted of 2,754 western AN people (1,218 men and 1,536 women). The age and gender distributions of the total WATCH cohort and the surveillance subset were similar (Table II). Baseline characteristics in the total WATCH cohort were compared with those in the follow-up surveillance subset (Figs. 2 and 3). Frequency of occurrence for categorical data and mean and dispersion for continuous data were also stratified by study to assess potential bias created by study consolidation. Although bias in 1 direction was not noted, the varied techniques for data acquisition may influence the analyses. Thus, notes were retained describing original study techniques.

## Discussion

The individual cohorts comprising the WATCH study provided cross-sectional prevalence data for the Norton Sound and Yukon-Kuskokwim regions. Each individual cohort had limited statistical power to assess incidence rates and examine associations between risk factors and chronic diseases. In contrast, the consolidated WATCH cohort provides a broader sample of western AN people and enhanced statistical power. This is the first study to enable analyses of rates of chronic diseases and risk factor associations in western AN people living in the remote, rural communities of the Norton Sound and Yukon-Kuskokwim regions.

Consolidation of the baseline data was aided by the similarity in the methods used by the 4 component studies. In cases where methods differed, the annotated variable keys will allow future investigators to analyze and interpret the combined data. Identical surveillance methods provide a resource for obtaining information on incidence rates of CVD and DM2 and assessing risk factors for each condition. This first phase of surveillance averaged 6.7 years; further follow-up will enhance the numbers of outcomes and allow more stratified analyses.

The WATCH investigators encouraged full participation of the participating communities. The Tribal Coordinating Committee provided advice and input in setting priorities for analyses of interest to the communities. A primary concern was confidentiality, leading to the double de-identification of the data in the final data set. As with studies in most Native communities, the data and samples belong to the community members; thus, the Tribal Coordinating Committee will continue to serve a key role in future data analyses and ancillary studies.

As in all collaborative studies, multiple IRBs and research review committees were involved, increasing the time required for initiation of the study and approvals for abstracts and manuscripts. Twenty-two months were required to obtain approval for data consolidation from all IRBs and tribal approval committees. This extended time can be viewed as a challenge to be met in developing a consortium of studies and obtaining funding. The investigators used this time to make decisions on variable consolidation methods and to design the database. By the time approval was granted, most of the procedures and database construction were in place.

The WATCH study's primary strength is the consolidation of 4 cohorts representative of populations in the 2 major areas of residency in western Alaska. Each component study adhered to standardized protocols with careful quality control. Thus, the combined baseline data set is of high quality, without large numbers of missing or outlier variables. The same surveillance and adjudication procedures were used for the component studies, assuring consistent outcome data. The Tribal Coordinating Committee assured input and acceptance by the communities, and all investigators were committed to adhering to the limits set by the communities. There are few examples of similar collaborations, and none have been conducted in an indigenous population. Three collaborations of studies have focused on White populations where data sets were harmonized to compare prevalence of risk factors (16) or to evaluate predictors of endpoints (17,18). Neither of the latter 2 used identical methods for follow-up. Three studies have analyzed prevalence data sets from more than 1 study of Inuit populations, but none of them involved prospective surveillance with common methods (19–21).

Although techniques were similar and bias was assessed, the analyses of combined variables may be less precise than the 4 original databases. Future investigators using WATCH data will need to consult the variable keys and consider how the data were collected and consolidated. While much larger than any 1 of its component studies, the number of outcomes for CVD and DM2 in this population remains small. Given the low rate of these outcomes and the relatively young age of the cohort, longer follow-up will increase the power and allow for additional stratified analyses.

In summary, a consolidated data set from 4 studies of western AN people has been created, allowing sufficient power to collect and analyze population-based data on CVD and DM2 in this population for the first time. This is an initial step in developing a translational research programme to reduce health disparities in western AN people.

## References

[1] Parran T, Ciocco A, Crabtree JA, McNerney WJ, McGibony JR, Wishik SM (1954). Alaska's health: a survey report to the United States Department of the Interior.

[2] Murphy NJ, Schraer CD, Theile MC, Boyko EJ, Bulkow LR, Doty BJ (1997). Hypertension in Alaska Natives: association with overweight, glucose intolerance, diet and mechanized activity. Ethn Health.

[3] Narayanan ML, Schraer CD, Bulkow LR, Koller KR, Asay E, Mayer AM (2010). Diabetes prevalence, incidence, complications and mortality among Alaska Native people, 1985–2006. Int J Circumpolar Health.

[4] Nobmann ED, Byers T, Lanier AP, Hankin JH, Jackson Y (1992). The diet of Alaska Native adults: 1987–1988. Am J Clin Nutr.

[5] Schraer CD, Ebbesson SO, Adler AI, Cohen JS, Boyko EJ, Nobmann ED (1998). Glucose tolerance and insulin-resistance syndrome among St. Lawrence Island Eskimos. Int J Circumpolar Health.

[6] State of Alaska Department of Labor and Workforce Development: Research and Analysis – 2010 census demographic profiles, 2010. http://live.laborstats.alaska.gov/cen/dparea.cfm.

[7] University of Alaska Fairbanks (n.d.). Alaska Native Language Center: Central Yup'ik. http://www.uaf.edu/anlc/languages/cy/.

[8] Lee ET, Cowan LD, Welty TK, Sievers M, Howard WJ, Oopik A (1998). All-cause mortality and cardiovascular disease mortality in three American Indian populations, aged 45–74 years, 1984–1988. The Strong Heart Study. Am J Epidemiol.

[9] Ebbesson SOE, Schraer CD, Risica PM, Adler AI, Ebbesson L, Mayer A (1998). Diabetes and impaired glucose tolerance in three Alaskan Eskimo populations: the Alaska Siberia project. Diabetes Care.

[10] Ebbesson SO, Ebbesson LO, Swenson M, Kennish JM, Robbins DC (2005). A successful diabetes prevention study in Eskimos: the Alaska Siberia project. Int J Circumpolar Health.

[11] Mohatt GV, Plaetke R, Klejka J, Luick B, Lardon C, Bersamin A (2007). The Center for Alaska Native Health Research Study: a community-based participatory research study of obesity and chronic disease-related protective and risk factors. Int J Circumpolar Health.

[12] Slattery ML, Schumacher MC, Lanier AP, Edwards S, Edwards R, Murtaugh MA (2007). A prospective cohort of American Indian and Alaska Native people: study design, methods, and implementation. Am J Epidemiol.

[13] Howard BV, Devereux RB, Cole SA, Davidson M, Dyke B, Ebbesson SOE (2005). A Genetic and Epidemiologic Study of Cardiovascular Disease in Alaska Natives (GOCADAN): design and methods. Int J Circumpolar Health.

[14] Alaska Native Health Board (n.d.). About ANHB. http://www.anhb.org/index.cfm?section=About.

[15] American Diabetes Association (2010). Standards of Medical Care in Diabetes – 2010. Diabetes Care.

[16] Meigs JB, Wilson PWF, Nathan DM, Agostino RB, Williams K, Haffner SM (2003). Prevalence and characteristics of the metabolic syndrome in the San Antonio Heart and Framingham Offspring studies. Diabetes.

[17] Balkau B, Shipley M, Jarrett RJ, Pyörälä K, Pyörälä M, Forhan A (1998). High blood glucose concentration is a risk factor for mortality in middle-aged nondiabetic men. Diabetes Care.

[18] Alonso A, Nettleton JA, Ix JH, de Boer IH, Folsom AR, Bidulescu A (2010). Dietary phosphorus, blood pressure, and incidence of hypertension in the Atherosclerosis Risk in Communities Study and the Multi-Ethnic Study of Atherosclerosis. Hypertension.

[19] Young TK, Bjerregaard P, Dewailly E, Risica PM, Jørgensen ME, Ebbesson SE (2007). Prevalence of obesity and its metabolic correlates among the circumpolar Inuit in 3 countries. Am J Publ Health.

[20] Bjerregaard P, Young TK, Dewailly E, Ebbesson SO (2004). Indigenous health in the Arctic: an overview of the circumpolar Inuit population. Scand J Public Health.

[21] Bjerregaard P, Dewailly E, Young TK, Blanchet C, Hegele RA, Ebbesson SE (2003). Blood pressure among the Inuit (Eskimo) populations in the Arctic. Scand J Public Health.

